# Long Non-coding RNA BTG3-7:1 and JUND Co-regulate *C21ORF91* to Promote Triple-Negative Breast Cancer Progress

**DOI:** 10.3389/fmolb.2020.605623

**Published:** 2021-01-29

**Authors:** Zheng Dan, He Xiujing, Luo Ting, Zhong Xiaorong, Zheng Hong, Yang Jiqiao, Li Yanchu, Jing Jing

**Affiliations:** ^1^Laboratory of Tumor Targeted and Immune Therapy, Clinical Research Center for Breast, West China Hospital, Sichuan University, Chengdu, China; ^2^Department of Head and Neck Oncology, West China Hospital, Sichuan University, Chengdu, China

**Keywords:** Triple-negative breast cancer, Lnc-BTG3-7:1, *C21ORF91* gene, GRB2-RAS-RAF-MEK-ERK pathway, GRB2-PI3K-AKT-GSK3β-β-catenin

## Abstract

**Background:**

Triple-negative breast cancer (TNBC) is a type of highly invasive breast cancer with poor prognosis. Recently, massive data reveal that long non-coding RNAs (lncRNAs) play important roles in cancer progress. Recently, although the role of lncRNAs in breast cancer has been well documented, few focused on TNBC. In this study, we aimed to systematically identify functional lncRNAs and to explore its molecular mechanism on TNBC progress.

**Methods:**

The recurrence of lncRNAs and their target genes were validated with TNBC biopsies and cell lines. Total one hundred and thirteen TNBC biopsies, including nineteen patient-matched samples, were collected. The profile of TNBC-related lncRNAs and their target genes were characterized by RNA sequencing (RNA-seq) and bioinformatic analysis. Tumor specific lncRNAs, which also showed biological function correlated with TNBC, were identified as potential candidates; and the target genes, which regulated by the identified lncRNAs, were predicted by the analysis of expression correlation and chromosome colocalization. Cross bioinformatic validation was performed with TNBC independent datasets from the cancer genome atlas (TCGA). The biological functions and molecular mechanism were investigated in TNBC model cell lines by cell colony forming assay, flow cytometry assay, western-blot, RNA Fluorescence *in situ* Hybridization assay (RNA FISH) and chromatin immunoprecipitation-qPCR (ChIP-qPCR).

**Results:**

Abundant Lnc-BTG3-7:1, which targets gene *C21ORF91*, was specifically observed in TNBC biopsies and cell lines. Knockdown of Lnc-BTG3-7:1 or *C21ORF91* strongly inhibited cell proliferation, promoted cell apoptosis and cell cycle G1-arrested. Meanwhile, investigation of molecular mechanism indicated that Lnc-BTG3-7:1, cooperated with transcription factor JUND, *cis*-regulated the transcription of *C21ORF91* gene, and down-regulation of Lnc-BTG3-7:1/*C21ORF91* suppressed GRB2-RAS-RAF-MEK-ERK and GRB2-PI3K-AKT-GSK3β-β-catenin pathways.

**Conclusions:**

In this study, we identified a TNBC specific lncRNA Lnc-BTG3-7:1, which sustained tumor progress. Up-regulation of Lnc-BTG3-7:1 promoted the transcription of oncogene *C21ORF91* and activated PI3K-AKT-GSK3β-β-catenin and MAPK pathways. Taken together, our results not only identified a biomarker for diagnosis but also provided a potential therapeutic target against TNBC.

## Introduction

Triple-negative breast cancer (TNBC) is the most aggressive breast cancer subtype and is characterized by poor survival (Shah et al., 2012; Johnson et al., 2020), occurring from younger age and being prone to distant metastasis. Moreover, it is kind of a heterogeneous disease with complex genetic background (Network, 2012), which represents different prognosis and responds to chemotherapies (Masuda et al., 2013; Burstein et al., 2015). However, current treatment outcomes are unsatisfied even under target therapy and immunotherapy, and unfortunately chemotherapy has to remain as the preferred treatment (Pandy et al., 2019). Thus, finding new target and understanding its mechanisms are essential. Recently, many studies report that long non-coding RNAs (LncRNAs) are likely pervasive in cancers and can drive cancers development, drug resistance and progress (Gupta et al., 2010; Martin and Chang, 2012; Bhan et al., 2017; Chi et al., 2019), which can be considered as important cancer biomarkers and play critical roles in cell proliferation, cell cycle, cell apoptosis, cell invasion, and metastasis (Yang et al., 2014; Li et al., 2016; Collina et al., 2019). Recently, although several lncRNAs in breast cancer have been well documented (Mourtada-Maarabouni et al., 2009), only a few of them focus on TNBC, such as MALAT1, LINK-A, HOTAIR, LINP1, LncRNA-RoR, Lnc-RNA BORG, LOC554202, and HULC (Loewer et al., 2010; Augoff et al., 2012; Tripathi et al., 2013; Shi et al., 2014; Tao et al., 2015; Jin et al., 2016; Lin et al., 2016; Zhang et al., 2016; Gooding et al., 2019), and the mechanisms are still unclear. Thus, potential TNBC specific lncRNAs are expected to be explored, and the molecular features of lncRNAs that underlie the development, metastasis, and relapse of TNBC remain to be elucidated.

Therefore, in this study, we aimed to systematically identify specific lncRNA by using RNA sequencing (RNA-seq), and to explore its functions and molecular mechanism on TNBC progress.

## Materials and Methods

### Patients’ Tissue Samples

Total one hundred and thirteen TNBC biopsies, including nineteen patient-matched samples, were collected from Breast cancer biobank of Western China Hospital. This experiment was approved by Ethics Committee of Western China Hospital.

### Cell Culture

MDA-MB-231, MDA-MB-468, BT-549 cells were used as representative human TNBC and MCF-10A cell was used as human mammary gland epithelial. MDA-MB-231, MDA-MB-468 were cultured by 10% fetal bovine serum in DMEM (Thermo Scientific HyClone, United States). BT-549 was cultured by 10% fetal bovine serum in RPMI 1640 Medium Modified (Thermo Scientific HyClone, United States). MCF-10A was cultured by 5% fetal horse serum (Solarbio, China) in DME/F-12 (Thermo Scientific HyClone, United States) which added with Hydrocortisone (0.5 μg/ml final, Sangon, Shanghai, China), insulin (10 μg/ml final, Novo Nordisk, Bagsvaerd, Denmark), EGF (20 ng/ml final, Sangon, Shanghai, China), cholera toxin (100 ng/ml final, MedChemExpress, New Jersey, United States). All culture mediums were added with 1% penicillin and streptomycin and the four cell lines were cultured at 37.0°C in an atmosphere of 5% CO_2_.

### RNA Extraction and High-Throughput Sequencing

TRIzol reagent (Invitrogen, Carlsbad, CA, United States) was used to isolated total RNAs from tissues and cells following the manufacture’s protocol. Library preparation and sequencing were performed as described in detail in our previous study (Deng et al., 2019). Briefly, RNA integrity and quality were assessed by 2100 Bioanalyzer RNA Nano Chip (Agilent Technologies, United States) and Nanodrop ND-2000 Spectrophotometer (NanoDrop Technologies, United States). Subsequently, library preparation was performed as described in NEBNext^®^ Ultra^TM^ Directional RNA Library Prep Kit for Illumina^®^ (NEB, United States) following manufacturer’s recommendations. The libraries were then sequenced on the Illumina Hiseq platform (Novogene Bioinformatic Technology Co., Ltd., China), following a 2 × 150-bp paired-end protocol. Sequencing data were available at the NCBI Sequence Read Archive under project number PRJNA553096.

### Sequencing Data Processing and LncRNAs Identification

After pre-processing filtering of low-quality reads and adaptor trimming, high-quality sequencing reads were aligned to the human reference genome assembly (GRCh37) using TopHat2 (Kim et al., 2013). Transcript assembly and abundance estimation were performed using Cufflinks (Trapnell et al., 2012) and HTSeq (Anders et al., 2015). The Ensembl, LNCipedia, and non-code databases were chosen as references to retrieve known transcripts. Novel transcripts were subjected to coding potential analysis by Coding-Non-Coding-Index (CNCI) (Sun et al., 2013), Coding Potential Calculator (CPC) (Kong et al., 2007), and Pfam-Scan (Chojnacki et al., 2017). The transcripts with lengths greater than 200 nt but without coding function were defined as candidate lncRNAs. To perform differential expression analysis in matching tumor and normal tissues, LPEseq (Gim et al., 2016) was utilized. Features with *q* value < 0.05 and | log2(Fold Change) | > 1 was considered differentially expressed.

### Target Gene Prediction and Function Analysis

The biological functions of differentially expressed lncRNAs were predicted via the positional relationship (cis-target) and expression correlation (trans-target) between lncRNAs and protein-coding genes. To classify lncRNAs *cis-*target genes, we searched protein-coding genes located in 100 kb upstream and downstream of differentially expressed lncRNAs. Protein-coding genes with absolute correlation value greater than 0.95 were considered to be *trans*-target genes. The interaction probability between lncRNAs and proteins was predicted by RPISeq (Muppirala et al., 2011). The functional analysis was conducted by KOBAS (Xie et al., 2011), and visualized by the clusterProfiler (Yu et al., 2012) and ggraph (Pedersen, 2017). To scrutinize how Lnc-BTG3-7:1 promotes tumor progress, co-expression network using the CEMiTool package (Russo et al., 2018) to identify functional protein interacted with its target gene *C21ORF91* were constructed. Protein-protein network (PPI) from STRING database (Szklarczyk et al., 2015) was further integrated into co-expression modules.

### Quantitative Real-Time PCR (qPCR)

The first strand complementary DNAs of the RNAs were synthesized using the ReverseAid First Strand cDNA Synthesis Kit (Thermo Fisher, United States). qPCR was performed using SYBR Green Supermix (Bio-Rad, United States). Lnc-BTG3-7:1 and *C21ORF91* expression in cell lines was normalized to β-actin using the comparative Ct method. Random primer was used for reverse transcription. Then, forward primer of Lnc-BTG3-7:1 and *C21ORF91*: TGC AACAACCCCATTTTTCCTA/GAACGTGTGCATGTGCTAAG and reverse primer: AAGAGTTCGGGCTCATCTCAC/TGAG TACCAGCACCACAAAG, respectively, were used to conduct qPCR.

### Short Hairpin RNA (shRNA) Synthesis and Transfection

Lnc-BTG3-7:1 and specific shRNAs were synthesized by Sangon Biotech (Shanghai, China). The shRNAs target Lnc-BTG3-7:1 and *C21ORF91* gene were shRNA241/shRNA351 and shRNA73/shRNA764, respectively. Sequences of shRNA241 and shRNA351 were: GCTGCTTTGTACTGATTGTAACTCGAGT TACAATCAGTACAAAGCAGCTTTTTG and GGTGCAGTT AACAGAGTTACGCTCGAGCGTAACTCTGTTAACTGCACC TTTTTG, respectively. The sequences of shRNA73 and shRNA764 were GGAGCAGTTTGTAAACATTGACTCGA GTCAATGTTTACAAACTGCTCCTTTTTG and GCAAAGCT CCTACAGCAAATCCTCGAGGATTTGCTGTAGGAGCTTTG CTTTTTG, respectively. These shRNAs were annealing and then inserted into the *pLKO.1* vector between the restriction sites *Age*I and *Eco*RI. The propagated synthetic construct vectors from *Escherichia coli* were extracted using Plasmid DNA purification kit (Macherey-Nagel, Germany). Lentiviral particles were produced by co-transfecting expression vector sh241_pLKO.1, sh351_pLKO.1, sh73_pLKO.1, and sh764_pLKO.1 with viral particle packaging helper vector into 293T cells. Tires of the four kinds of viral particles were determined by limited serial dilution. The three TNBC cell lines were infected with the four kinds of packaged lentivirus. The efficiency of Lnc-BTG3-7:1 and *C21ORF91* single or double knockdown were determined by qPCR.

### Cell Proliferation Assay

Mock and Lnc-BTG3-7:1/*C21ORF9*1 gene knockdown TNBC cells (MDA-MB-231, MDA-MB-468, and BT-549) were seeded in 35 mm dish with 2 mm grid at 1 × 10^5^ cells/dish, respectively. Then, we recorded cell growth curve after culturing for 0, 24, 72, and 96 h to determine short-term cell proliferation capacity.

### Colony Forming Assay

Mock and Lnc-BTG3-7:1/*C21ORF91* gene single or double knockdown TNBC cells were seeded in 12-well plate at 2 × 10^3^ cells/well for MDA-MB-231 and MDA-MB-468 cells, and 1 × 10^3^ cells/well for BT-549 cells, respectively. Then, cells were cultured for continuous 8 days. After fixation with 4% paraformaldehyde for 30 min, cells were stained with crystal violet solution for 30 min.

### Western-Blot Assay

Expression of ERK (#4695, CST, United States), p-ERK (#4377, CST, United States), MEK (#4694, CST, United States), p-MEK (#9154, CST, United States), AKT (#9272, CST, United States), AKT-Ser473 (#9271, CST, United States), β-catenin (#9582, CST, United States), c-MYC (#9402, CST, United States) and GSK-3β(#9315, CST, United States) were measured by Western-blot in MDA-MB-231, MDA-MB-468 and BT-549. Each 10 μg of protein was resolved on SDS-PAGE and transferred to PVDF membranes (Millipore, United States). Immunoblotting was performed overnight at 4°C. The membranes were then washed with Tris Buffered saline Tween (TBST) three times and incubated with the corresponding secondary antibodies (1:5000) at room temperature for 1 h. Then, these membranes were washed with TBST for three times. Images were captured by Bio-Rad ChemiDoc MP (Bio-Rad, United States).

### Flow Cytometry Assay

Mock and Lnc-BTG3-7:1/*C21ORF9*1 gene single or double knockdown TNBC cells (MDA-MB-231, MDA-MB-468, and BT-549) were trypsinized and resuspended to obtain single–cell suspensions, respectively. (1) Cells for cell-cycle analysis were fixed in ice-cold 70% ethanol overnight, and stained with propidium iodide (5 μg/mL) and RNase A (50 units/mL). (2) Cells for apoptosis analysis were stained with fluorescein isothiocyanate-conjugated Annexin V and propidium iodide (Apoptosis Detection kit; 4A Biotech) for 15 min as recommended by the manufacturer. Samples were tested by CytoFLEX flow cytometry (Beckman, CA, United States) and analyzed by FlowJo V10.0 software (Becton Dickinson, Ashland, OR, United States).

### RNA Fluorescence *in situ* Hybridization Assay (RNA FISH)

An oligonucleotide probe that was complementary to Lnc-BTG3-7:1 (designed by Stellaris Designer) and labeled with Cy3 dye at 5’ was purchased from Sangon Biotech (Shanghai, China). A Cy3-labeled sense oligonucleotide was used as negative control. The sequences of the lncRNA probe and negative control (NC) probe were as follows: CCCAGTCAACACTCATACTT, CCATCCTATACCAATCTCGA, respectively. MDA-MB-231 and BT-549 cells were collected at exponential growth phase and resuspended to obtain single-cell suspensions (5 × 10^4^/ml). Then, cells were seeded into multi-chambered coverglass slides and cultured at normal growth conditions overnight. The cells were fixed in 4% formaldehyde for 15 min after washing 5 min for 3 times by phosphate buffer saline (PBS). Then, cells were treated with RNA FISH Kit (Fluorescent *in situ* Hybridization Kit, RiboBio, China) as recommended by the manufacturer, and Lnc-BTG3:7:1 probe and NC probe were cultured at 0.5 μM in 37°C overnight. Then, slides were incubated with JUND antibody (1:400, #5000S, CST, United States) for 2 h and incubated with the corresponding secondary fluorescence antibody (FITC, 1:200, Invitrogen, United States) for 40 min at room temperature after washing by PBS for three times. Last, the slides were sealed with DAPI Fluoromount-G (Yeasen Biotech, China). Images were captured by laser scanning confocal microscope (Nikon, Japan) and analysis with software NIS-Elements BR (Version 5.11.01).

### Chromatin Immunoprecipitation (ChIP)-qPCR

ChIP assays (Jing et al., 2020) were performed for the MDA-MB-231, MDA-MB-468, and BT-549 cells. Cells (approximately 1 × 10^7^ cells) were digested into cell suspension using trypsin and washed with PBS. Then crosslink was performed using 3% methanol and quenched by 0.125 M glycine. The cells were rinsed and scraped off into conical tubes. Every 0.1 g pellets were resuspended in 1 mL cell lysis buffer and then sonicate (SCIENTZ-II D, Ningbo Scientz Biotechnology, Zhejiang, China) until the cell lysis turned clear to fragmentate chromatin to desired size (100–500 bp). The sonicated lysates were diluted using RNA dilution buffer with RNase inhibitor. After pre-hybridization, JUND antibody (#5000S, CST, United States) and 40 μL of beads were added together for immunoprecipitation. IgG was used as negative control. The RNA–protein complex was eluted, reverse crosslinked, and purified for qPCR and western-blot, respectively.

### Statistical Analysis

The level of significance was defined as *P* < 0.05. The difference between mean values was assessed by *t*-test using Prism GraphPad 8.0 (GraphPad Software, United States).

## Results

### Characterization of LncRNA and Transcriptome of TNBC

**FIGURE 1:**
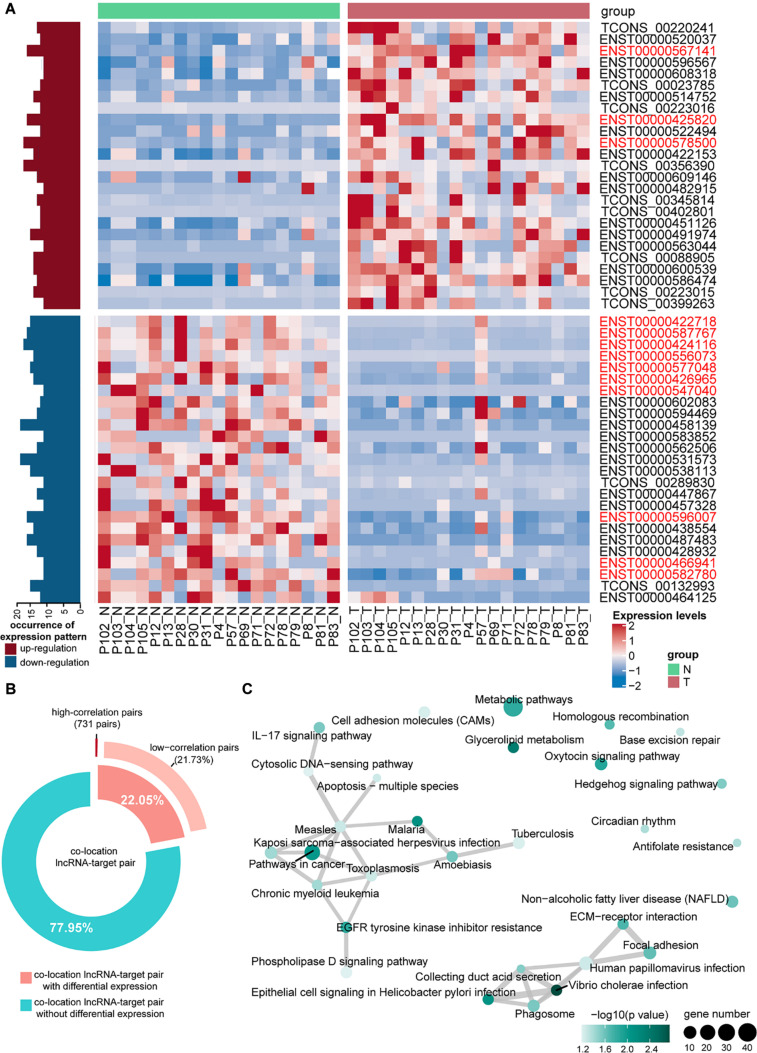
Characterization and functional analysis differentially expressed LncRNAs (DE-lncRNAs) and differentially expressed target genes (DEGs). **(A)** Heatmap with expression characteristics of differentially expressed lncRNAs in tumor and normal tissues. The order of DE-lncRNAs was determined by occurrence in comparison group. The top 50 most differentially expressed lncRNAs used for clustering were plotted, of which 25 were up-regulated and 25 down-regulated, respectively. The occurrence of up-regulated and down-regulated patterns were shown as red and blue bars on the left side. Samples were also annotated on top. The DE-lncRNAs verified in TCGA TNBC cohorts were highlighted in red. **(B)** LncRNA target prediction analysis based on positional relationship and expression correlation (as described in “Materials and Methods”). **(C)** Pathway enrichment analysis of target genes differentially expressed lncRNAs. The relationships of important biological processes (top 30) were depicted by network. The color was scaled by *P*-values, and the sizes of the dots represented the percentage of each term.

According to the specific structural and non-coding features of lncRNA, 58,163 lncRNAs were identified in at least one sample. Of these, 48,551 (83.47%) were identified as known lncRNAs, and 9,612 novel lncRNAs were detected in TNBC tissues. To investigate the key lncRNAs involved in TNBC progress, the expression profiles of lncRNAs between tumor and normal tissues were compared in order to detect the differentially expressed lncRNAs (DE-lncRNAs). We identified 864 lncRNAs with high occurrence (occurrence >10) displayed differential expression in TNBC biopsies. Of the dysregulated lncRNAs, 193 lncRNAs had consistent expression patterns in TNBC biopsies. Among them, 62 lncRNAs were highly expressed in tumor tissues, whereas, 131 lncRNAs were down-regulated, compared with normal tissues. Several DE-lncRNAs with high occurrence were further verified in TCGA TNBC cohorts (Figure 1A).

To better understand the role of lncRNAs in TNBC progress, functional analysis of target genes was performed to predict the biological functions of DE-lncRNAs. A total of 671 protein-coding genes were significantly correlated with neighbor lncRNAs (Figure 1B). We then conducted pathway analysis to gain insight into the functions of DE-lncRNA target genes, and found that the target genes of DE-lncRNAs were highly enriched in pathways in cancer (Figure 1C). Moreover, numerous target genes were involved in oncogenic signaling pathways, such as PI3K-AKT signaling pathway (12 target genes), RAS signaling pathway (9 target genes), and MAPK signaling pathway (7 target genes). To find out candidate lncRNAs, we removed DE-lncRNAs whose occurrence less than 10 prior to subsequent analyses (Figure 1C).

### Lnc-BTG3-7:1 and Target Gene *C21ORF91* Were Identified in TNBC Specific Pattern

**FIGURE 2:**
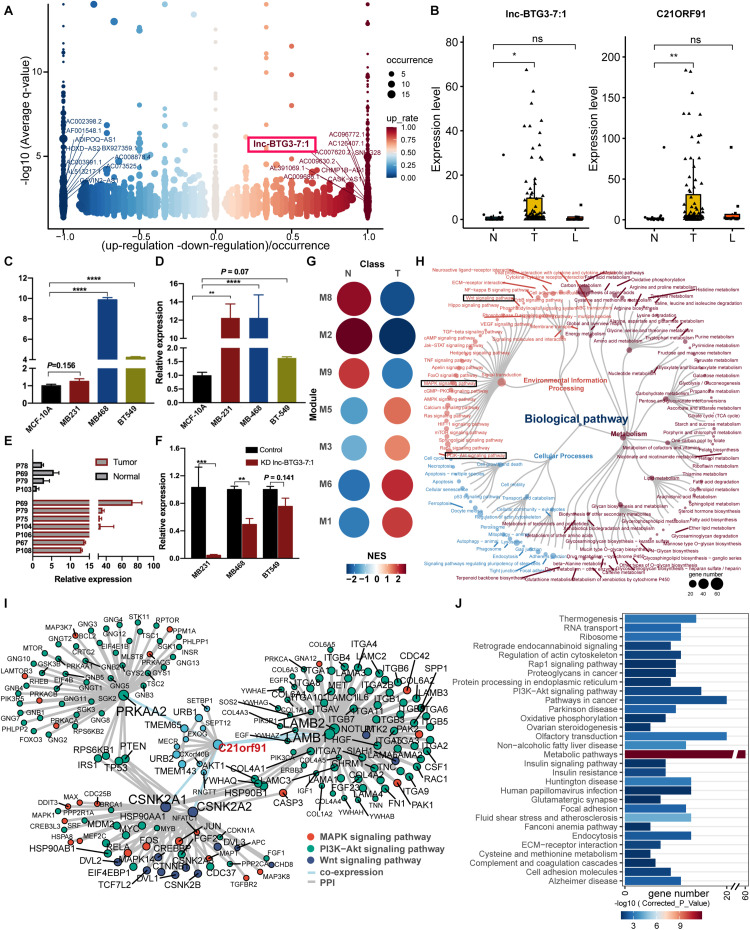
Identification of functional LncRNA and targeted genes potentially driving TNBC progress. **(A)** Differential expression of lncRNAs in TNBC biopsies. DE-lncRNAs were ranked by fold change and occurrence. The ten up- and down-expressed lnc-RNAs were labeled. Lnc-BTG3-7:1 was highlighted with a red frame in the up-regulated group. **(B)** The expression level of Lnc-BTG3-7:1 in TNBC biopsies (*n* = 113) was higher than normal breast tissue (*P* < 0.005***), accompanied by the *C21ORF91* gene expression increased (*P* < 0.005***). **(C,D)** The abundance of Lnc-BTG3-7:1 **(C)** and *C21ORF91* **(D)** highly increased in TNBC cells compared to MCF10A (*P* < 0.05). **(E)** A total of seven TNBC tumor tissue samples and four normal breast tissue samples were tested by qPCR, and a remarkably higher expression of Lnc-BTG3-7:1 in tumor tissues was showed compared to normal tissues (*P* < 0.005***). **(F)** Knockdown of lnc-BTG3-7:1 suppressed the *C21ORF91* gene expression in MDA-MB-231 (*P* < 0.005***) and MDA-MB-468 (*P* < 0.005**), respectively. **(G)** Gene set enrichment analyses showing the module activity in tumor tissues and adjacent normal tissues. Point size and color represented the normalized enrichment score (NES). **(H)** Functional annotation based on KEGG functional hierarchy for genes in module M3. Node size represented the number of genes or children terms. Node color indicated the functional classification of pathways. **(I)** Interaction network suggested a pivotal role of Lnc-BTG3-7:1 and *C21ORF91* in TNBC progress via coordinating oncogenic signaling. The genes involved in Wnt/β-catenin, Ras/MAPK, and PI3K/AKT pathways were color-marked. **(J)** Over-representation analysis of co-expressed genes of *C21ORF91*. The bar chart showed the gene number in the corresponding pathway. The color of the bar indicated the statistical significance. *P < 0.05 and ***P < 0.001.

To predict and identify the potential TNBC specific lncRNAs, DE-lncRNAs were ranked by fold change and occurrence. The top-ranked genes were defined as TNBC specific lncRNAs. Among them, Lnc-BTG3-7:1 was eventually screened out using text mining and highlighted in Figure 2A. The expression levels of Lnc-BTG3-7:1 and its target gene *C21ORF91* were both significantly different between tumor and normal tissue (Pearson product-moment correlation coefficient >0.97; *P*-value < 2.2e-16, Figure 2B). Then, the specific expression patterns of Lnc-BTG3-7:1 and *C21ORF91* in TNBC samples were further detected in TNBC cells and normal human breast epithelial cell line by qPCR and Western-blot. The results suggested that Lnc-BTG3-7:1 highly expressed in MDA-MB-468 and BT-549 cells compared with MCF-10A (*P* < 0.05****, Figure 2C), but invalid in MDA-MB-231 (*P* = 0.16, Figure 2C). Meanwhile, *C21ORF91* protein expression was significantly higher in MDA-MB-231 (*P* < 0.05**, Figure 2D) and MDA-MB-468 (*P* < 0.05****, Figure 2D), respectively, compared to MCF-10A cells, but invalid in BT-549 cells (*P* = 0.07, Figure 2D). Meanwhile, the expression of Lnc-BTG3-7:1 was verified in patients’ tissue samples by using qPCR, and the same expression trend was observed consistently with RNA-seq (Figure 2E). Furthermore, in order to investigate whether Lnc-BTG3-7:1 targets *C21ORF91* gene, we knocked down the Lnc-BTG3-7:1 in MDA-MB-231, MDA-MB-231, and BT-549 cell lines, and the result showed that after knocking down Lnc-BTG3-7:1, the *C21ORF91* expression in TNBC cell lines significantly and synchronously decreased (*P* < 0.05, Figure 2F).

To address the question of how Lnc-BTG3-7:1 function in concert with target gene *C21ORF91* to regulate TNBC progress, the possible interaction network was constructed. We then concentrated on module M3, which contained *C21ORF91*. Gene set enrichment analyses showed module M3 had higher activity in tumor tissues (Figure 2G). Remarkably, not only genes in module M3 participated in signal pathway (Figure 2H), and directly related to Wnt/β-catenin, RAS/MAPK, and PI3K/AKT pathways (Figure 2I), but also functional enrichment analysis revealed that co-expressed genes of *C21ORF91* were substantially enriched in PI3K/AKT pathway (Figure 2J). The results indicated that Lnc-BTG3-7:1 and *C21ORF91* gene might involve in Wnt/β-catenin, RAS/MAPK and PI3K/AKT pathways.

### Lnc-BTG3-7:1 Participates in Regulating TNBC Progress

**FIGURE 3:**
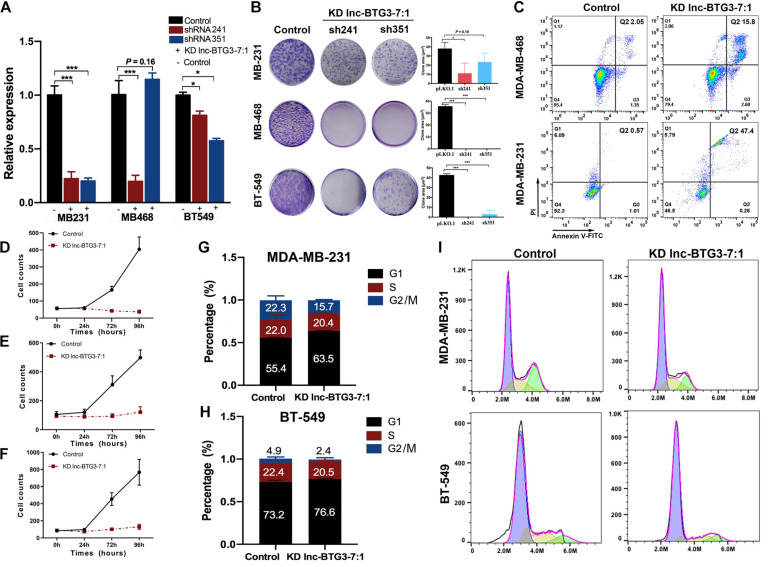
Lnc-BTG3-7:1 participates in the process of TNBC progress. **(A)** The expression level of Lnc-BTG3-7:1 in three TNBC cell lines significantly decreased after Lnc-BTG3-7:1 knockdown (KD). KD efficiency of shRNA 351 was relatively poor in MDA-MB-468. (*P < 0.05, ***P < 0.005). **(B)**. Knockdown of Lnc-BTG3-7:1 significantly reduced colony forming capacity of the three TNBC cell lines (*P* < 0.05). **(C)** Knockdown of Lnc-BTG3-7:1 (with shRNA241) induced early apoptosis (Q3: 2.68%) cells and late apoptosis (Q2: 15.8%) cells compared to that of the control group (Q3: 1.35%, Q2: 2.05%) in MDA-MB-468 cell, and the early apoptosis (Q3: 0.26%) cells and late apoptosis (Q2: 47.4%) cells compared with the control group (Q3: 1.01%, Q2: 0.57%) in MDA-MB-231 cells, respectively (*P* < 0.05). **(D–F)** Short-term proliferation capacity of three TNBC cell lines was significantly inhibited in KD Lnc-BTG3-7:1group (*P* < 0.05). **(G–I)** Cell cycle was arrested in G1 phase both in MDA-MB-231 and BT-549 in KD Lnc-BTG3-7:1 group (*P* < 0.05).

Lnc-BTG3-7:1 is a TNBC specific lncRNA, and its function still undefined. Thus, to investigate the function of Lnc-BTG3-7:1, we knocked down Lnc-BTG3-7:1 by using shRNA241 and shRNA351, and the knockdown efficiency of shRNA241 was significantly obvious (*P* < 0.05, Figure 3A). By performing colony forming assay, the long-term proliferation capacity of three TNBC cells was suppressed after knocking down Lnc-BTG3-7:1. The inhibition rates by shRNA241 were 82.1 ± 3.0%, 100 ± 4.5%, 100 ± 3.9%, and the inhibition rates by shRNA351 were 38.5 ± 8.5%, 100 ± 4.5% and 93.8 ± 7.9% in MDA-MB-231, MDA-MB-468, and BT-549, respectively (*P* < 0.05***, Figure 3B).

Next, we investigated the effects of Lnc-BTG3-7:1 on cell apoptosis. As shown in Figure 3C, the proportion of Annexin V (+)/PI (−) and Annexin V (+)/PI (+) apoptotic cells increased significantly after knocking down Lnc-BTG3-7:1 by shRNA241 (*P* < 0.05). Meanwhile, the short-term proliferation capacity of TNBC cells was also significantly suppressed in Lnc-BTG3-7:1 knockdown group. The inhibition rates were 94.8 ± 23.2%, 90.7 ± 6.1%, 94.3 ± 22.7% in MDA-MB-231, MDA-MB-468 and BT-549, respectively (*P* < 0.05***, Figures 3D–F). Additionally, knockdown of Lnc-BTG3-7:1 by shRNA241 arrested cell cycle in G1 phase in MDA-MB-231 (55.4 vs. 63.5%, *P* < 0.05***, Figures 3G,I) and BT-549 (73.2 vs. 76.6%, *P* < 0.05***, Figures 3H,I). Altogether, the results indicated that Lnc-BTG3-7:1 could promote TNBC progress.

### Lnc-BTG3-7:1 Target *C21ORF91* Gene to Regulate TNBC Progress

**FIGURE 4:**
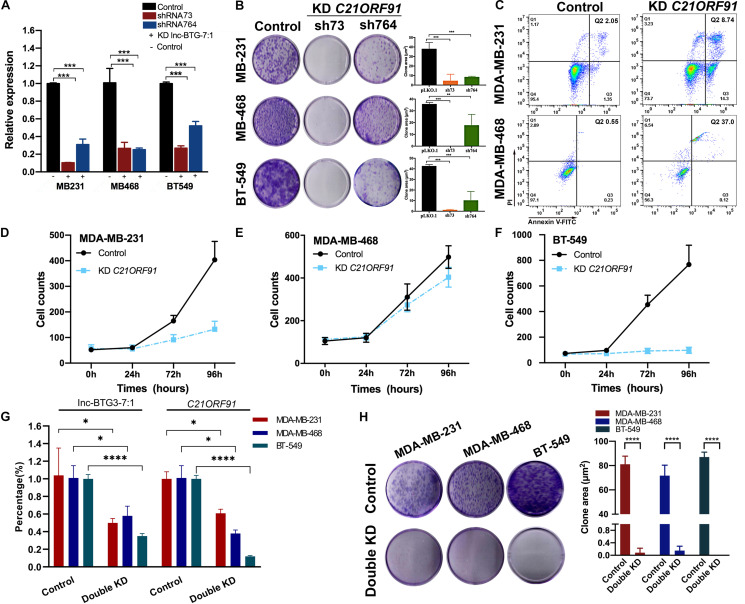
Lnc-BTG3-7:1 and target *C21ORF9*1 gene participates in the process of TNBC progress. **(A)** The expression levels of *C21ORF91* in three TNBC cell lines significantly decreased after *C21ORF91* gene knockdown (*P < 0.05, **P < 0.01, ***P < 0.005). **(B)** Knockdown of *C21ORF91* significantly reduced colony forming capacity of the three TNBC cell lines (*P* < 0.05). **(C)** Knockdown of *C21ORF91* (with shRNA73) induced early apoptosis (Q3: 14.3%) cells and late apoptosis (Q2: 8.74%) cells compared to that of the control group (Q3: 1.35%, Q2: 2.05%) in MDA-MB-468; and early apoptosis (Q3: 0.12%) cells and late apoptosis (Q2: 37.0%) cells compared with the control group (Q3: 0.23%, Q2: 0.55%) in MDA-MB-231 (*P* < 0.05), respectively. **(D–F)** Short-term proliferation capacity of TNBC cell lines were inhibited after knocking down of *C21ORF91*, especially in MDA-MB-231 and BT-549 (*P* < 0.05). **(G)** The expression levels of *C21ORF91* in three TNBC cell lines significantly decreased after double knockdown of Lnc-BTG3-7:1 and *C21ORF91* gene (*P < 0.05, **P < 0.01, ***P < 0.005). **(H)** Double knockdown of Inc-BTG3-7:1 and *C21ORF91* gene inhibited long-term proliferation capacity of three TNBC cells (*P* < 0.05****).

In previous study, few results showed that *C21ORF91* gene was involved in hepatocellular carcinoma cells (HCC) progress, and no report of *C21ORF91* gene focus on breast cancer, especially TNBC. Thus, to investigate the functions of *C21ORF91* gene in TNBC, we knocked down the expression of *C21ORF91* gene using shRNA73 and shRNA764, and the knockdown efficiency of shRNA73 was significantly obvious (*P* < 0.05***, Figure 4A). The result of colony forming assay showed that knockdown of *C21ORF91* gene could also inhibit long-term proliferation capacity. The inhibition rates by shRNA73 were 88.9 ± 17.8%, 100 ± 4.5%, 97.7 ± 1.9%, and the inhibition rates by shRNA764 were 78.2 ± 16.3%, 50.6 ± 23.1%, 76.5 ± 16.5% in MDA-MB-231, MDA-MB-468 and BT-549, respectively (*P* < 0.05***, Figure 4B).

Next, we investigated the effects of *C21ORF91* gene on cell apoptosis and proliferation. As shown in Figure 4C, the proportion of Annexin V (+)/PI (−) and Annexin V (+)/PI (+) apoptotic cells significantly increased after knocking down *C21ORF91* gene by shRNA73 compared to control group (*P* < 0.05). Meanwhile, short-term proliferation capacity of three TNBC cells was also significantly suppressed in *C21ORF91* gene knockdown group. The inhibition rates were 78.2 ± 22.6%, 30.8 ± 8.8%, 95.2 ± 23.4% in MDA-MB-231, MDA-MB-468, and BT-549, respectively (*P* < 0.05***, Figures 4D–F). Altogether, above results indicated that *C21ORF91* gene was involved in TNBC progress.

To further investigate the co-regulate effects of Lnc-BTG3-7:1 and *C21ORF91* gene on anti-TNBC function, Lnc-BTG3-7:1 and *C21ORF91* gene were double knocked down by using shRNA241 and shRNA73 in MDA-MB-231, MDA-MB-468, and BT-549. According to the result, double knockdown of Lnc-BTG3-7:1 and *C21ORF91* gene inhibited long-term proliferation capacity. The inhibition rates were 99.9 ± 0.14%, 99.8 ± 0.15%, 100 ± 0% in MDA-MB-231, MDA-MB-468 and BT-549, respectively (*P* < 0.05****, Figures 4G,H). Thus, the result showed that Lnc-BTG3-7:1 and *C21ORF91* gene co-regulated and involved in TNBC progress.

### Lnc-BTG3-7:1 and JUND Co-regulate the Transcription of *C21ORF91* in TNBC

**FIGURE 5:**
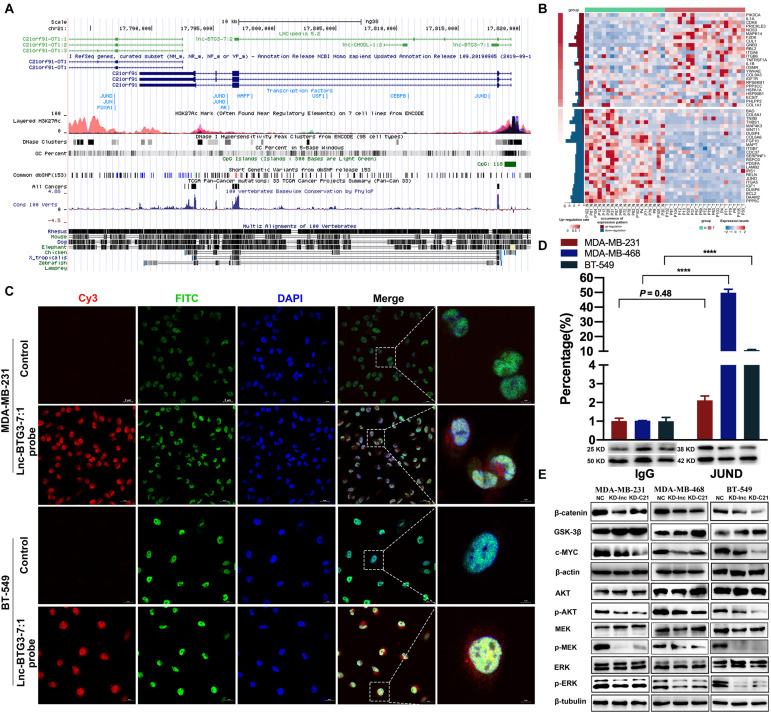
Lnc-BTG3-7:1 and JUND co-regulate the transcription of *C21ORF91* by activating AKT-GSK3β-β-catenin and MAPK pathways in TNBC. **(A,B)** Bioinformatic analysis shown JUND and Lnc-BTG3-7:1 had high possibility of interaction. **(C)** RNA fluorescence *in situ* hybridization and immunofluorescence co-localization analysis. The probes of control and Lnc-BTG3-7:1 was visualized in TNBC cells MDA-MB-231 (upper panel) and BT549 (lower panel). **(D)** ChIP-qPCR. Lnc-BTG3-7:1 was enriched in JUND specific antibody immunoprecipitation group in MDA-MB-231 (*P* = 0.48), MDA-MB-468 (*P* < 0.05****) and BT-549 (*P* < 0.05****) cells, respectively. **(E)** After knocking down Lnc-BTG3-7:1 or *C21ORF91* gene, the expression of *p*-AKT, β-catenin and c-MYC proteins decreased and was along with GSK-3β expression increased in GRB2-PI3K-AKT-GSK3β-β-catenin pathway, at the same time, the expression of *p*-ERK and *p*-MEK in GRB2-RAS-RAF-MEK-ERK pathway decreased.

No previous study has reported the mechanism of how Lnc-BTG3-7:1 regulates *C21ORF91*. To investigate the underlying mechanism, analysis of lncRNA-protein interaction was used to predict the potential way, and the prediction showed that Lnc-BTG3-7:1 might be synergistic with JUND to co-regulate target genes through *cis*-acting SE-lncRNA mechanism (Figures 5A,B). Furthermore, in order to confirm the prediction, RNA-FISH and immunofluorescence co-localization analysis were performed to determine the sub-location of Lnc-BTG3-7:1 and co-localization with JUND protein in MDA-MB-231 and BT-549 cells. The location of Lnc-BTG3-7:1, which was mainly in nucleus of TNBC cells and co-located with JUND protein, was observed (Figure 5C). More than that, we further performed ChIP-qPCR (Immunoprecipitation-qPCR), and significant enrichment of Lnc-BTG3-7:1 in JUND specific antibody immunoprecipitation group in MDA-MB-468 (*P* < 0.05****) and BT-549 (*P* < 0.05****) cells were observed compared with the negative control group (Figure 5D), which confirm the combination of Lnc-BTG3-7:1 and JUND. Thus, these results further showed that Lnc-BTG3-7:1 co-regulated with JUND in TNBC.

Altogether, based on the results, we found that Lnc-BTG3-7:1 was synergistic with JUND in the TNBC cells and potentially co-regulated the transcription of *C21ORF91*.

### Lnc-BTG3-7:1/*C21ORF91* Activated AKT-GSK3β and MAPK Pathways in TNBC

Previous report indicated that GRB2 is one of *C21ORF91* potential target proteins (Wang et al., 2008). In line with this conclusion, we also found that *C21ORF91* was involved in regulation of GRB2-RAS-RAF-MEK-ERK and GRB2-PI3K-AKT-GSK3β-β-catenin pathways (The data were shown in Figure 2H). To validate the activation of GRB2 signal, we checked the downstream kinase of MAPK and PI3K-AKT-GSK3β pathways. On the one hand, the result indicated that the phosphorylation of AKT at serine 473 a.a. residue was suppressed upon *C21ORF91* and Lnc-BTG3-7:1 knockdown in MDA-MB-231, MDA-MB-468 and BT-549 cell lines (Figure 5E). The suppression of p-AKTs473 resulted in hyper phosphorylation of GSK3beta, and subsequentially, down regulation of beta-catenin and c-myc. On the other hand, removal of *C21ORF91* or Lnc-BTG3-7:1 inhibited the downstream of canonical MAPK kinases MEK1/2 and ERK1/2 (Figure 5E).

**FIGURE 6:**
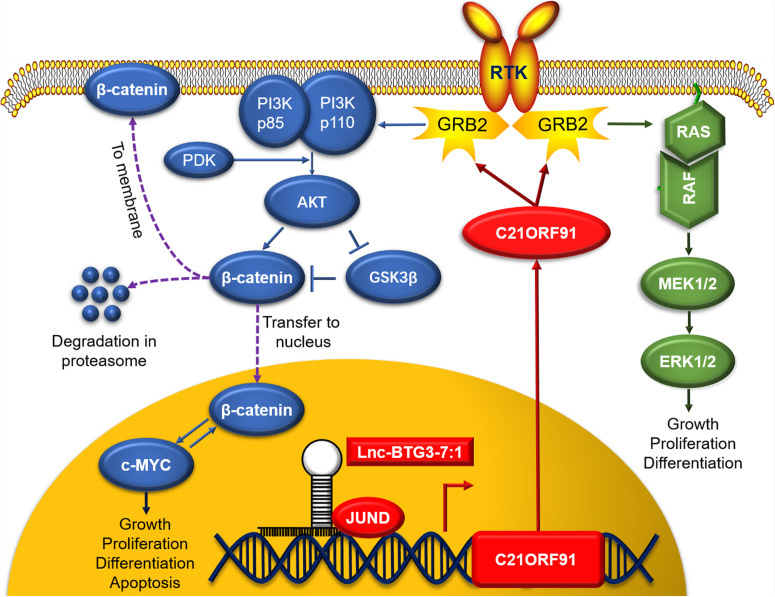
In nucleus, Lnc-BTG3-7:1 co-regulates the transcription of *C21ORF91* gene with nuclear synergistic transcription factor JUND. In cytoplasm, *C21ORF91* protein regulated AKT phosphorylation, β-catenin, c-MYC and GSK-3β in GRB2-PI3K-AKT-GSK3β-β-catenin pathway and activated ERK phosphorylation in GRB2-RAS-RAF-MEK-ERK pathway.

In summary, the potential working model is describe as: (1) JUND along with Lnc-BTG3-7:1 transcriptional upregulates *C21ORF91*; (2) *C21ORF91* translocates from nucleus to cytosol and activates GRB2; (3) GRB2 activates multiple pathways including PI3K and MAPK; (4) upon activation, PI3K signals downstream AKT and GS3Kbeta which in turn stabilizes b-catenin; (5) similarly, GRB2 activated RAS signals the canonical MAPK cascade, including RAF, MEK, ERK; and (6) these activated kinases promote the proliferation and anti-apoptosis of TNBC (Figure 6).

## Discussion

Triple-negative breast cancer is a high invasive with poor prognosis subtype breast cancer, occurring from younger age and being prone to distant metastasis (Foulkes et al., 2010; Shah et al., 2012). Based on the National Comprehensive Cancer Network guideline (NCCN), there is few clinical success of current therapy, making most TNBC patients have to still rely on chemotherapy (Bianchini et al., 2016; Park et al., 2018), and the median overall survival (mOS) for advanced TNBC patients are only about 9–12 months (Belli et al., 2019). Meanwhile, identifying effective biomarkers for cancer prognosis and drug responsiveness is of great importance in improving the clinical management of cancer, but unfortunately, precise biomarkers of TNBC treatment response and prognosis have not yet been identified (Singh et al., 2010).

Recently, the whole genome and transcriptome sequencing technology have been widely used in discovering latest genome difference (Marotti et al., 2017), which indicate that lncRNAs contribute to a significant portion of the “dark matter” of the human transcriptome (Kapranov et al., 2010) and show a new insight into the regulator heterogeneity of TNBC. Meanwhile, the differential expression of lncRNAs between normal and tumor tissues suggests that it is similar to protein-coding oncogenes (Huarte and Rinn, 2010), and dysfunction of lncRNA strongly associates with cancer progress (Fang et al., 2020). For example, UCA1, LncRNA human ovarian cancer-specific transcript 2 (HOST2) and LncRNA nuclear enriched abundant transcript1 (NEAT1) are associated with lung cancer, ovarian cancer and prostate cancer, respectively (Chakravarty et al., 2014; Gao et al., 2015; Nie et al., 2016). Nowadays, several lncRNAs have been shown to modify critical breast cancer associated molecular pathways such as GAS5 (Mourtada-Maarabouni et al., 2009). However, so far, there is rare outstanding results concerning TNBC in the field of lncRNAs (Gooding et al., 2019; Zhang et al., 2019), such as HOTAIR, which relates with luminal androgen receptor (LAR) subtype of TNBC (Gupta et al., 2010; Collina et al., 2019).

Also, previous studies have indicated that the *C21ORF91* gene encoding cytosolic protein plays a role in biological processes, such as cerebral cortex neuron differentiation, cell differentiation and regulation of dendritic spine development (Danileviciene et al., 2019). Recently, few researches show relationship between the *C21ORF91* gene and herpes labialis, in which *C21ORF91* gene is identified as direct targets of miR-194 in hepatocellular carcinoma cells (HCC) (Kriesel et al., 2011; Bao et al., 2015), but its function is not clear. The way in which the *C21ORF91* gene influences TNBC progress has not been established yet, and how Lnc-BTG3-7:1 regulates *C21ORF91* gene is still unknown.

In this study, we first screened out TNBC specific lncRNA Lnc-BTG3-7:1, which sustained tumor progress, and this TNBC specific lncRNA and its target *C21ORF91* gene were involved in Wnt/β-catenin and MAPK pathways, which were associated with cancer cell progress (Liu et al., 2019). According to the bioinformatic analysis, the 1–195th bases of the Lnc-BTG3-7:1 sequence overlapped with the 25–219th bases of the *C21ORF91* and the coding DNA sequence (CDS) of *C21ORF91* started from the 62nd base. Meanwhile, based on analysis of the gene sequence of *C21ORF91*, we not only found that Lnc-BTG3-7:1 located in the region of super enhance SE_13873\SE_18618, which belonged to *cis*-acting SE-lncRNA, but also lncRNA-protein interaction prediction showed that JUND and Lnc-BTG3-7:1 had high possibility of interaction. Thus, we predicted that Lnc-BTG3-7:1 might be synergistic with JUND to co-regulate target genes through SE-LncRNA mechanism, which was associated with Wnt/β-catenin and MAPK pathways.

Furthermore, in this study, we observed the functions of the Lnc-BTG3-7:1 and found its regulation mechanism in TNBC cell lines. (1) In functional analysis, single or double knockdown of Lnc-BTG3-7:1 and *C21ORF91* gene, strongly induced diminishing of cell proliferation, increasing cell apoptosis and arresting cell cycle in G1. (2) In mechanism analysis, by using IF/FISH and ChIP-qPCR assay, not only Lnc-BTG3-7:1 was co-located with JUND in nucleus, but also Lnc-BTG3-7:1 was enriched in JUND specific antibody immunoprecipitation groups in TNBC cell lines. Taken together, our result demonstrated that Lnc-BTG3-7:1 co-regulate the transcription of *C21ORF91* gene with nuclear synergistic transcription factor JUND. Meanwhile, Western-blot test showed that Lnc-BTG3-7:1 and the target *C21ORF91* gene were involved in GRB2-PI3K-AKT-GSK3β-β-catenin and GRB2-RAS-RAF-MEK-ERK pathways in TNBC.

Thus, from the above, our findings suggested that Lnc-BTG3-7:1 and the target *C21ORF91* gene were TNBC specific factors and participated in TNBC progress.

## Conclusion

In our study, a TNBC specific lncRNA Lnc-BTG3-7:1 was screened out and verified, which was involved in TNBC progress and could activate PI3K-AKT-GSK3β and MAPK pathways by regulating transcription of the target *C21ORF91* gene. In conclusion, our results not only identified Lnc-BTG3-7:1 as a biomarker for diagnosis, but also provided a potential therapeutic target against TNBC.

## Data Availability Statement

The datasets presented in this study can be found in online repositories. The names of the repository/repositories and accession number(s) can be found below: https://www.ncbi.nlm.nih.gov/, PRJNA553096.

## Ethics Statement

The studies involving human participants were reviewed and approved by the Ethics Committee of West China Hospital of Sichuan University. Written informed consent for participation was not required for this study in accordance with the national legislation and the institutional requirements.

## Author Contributions

LY and JJ: conceptualization. ZD and HX: formal analysis. ZD, HX, LT, ZX, and YJ: methodology. ZD, LY, JJ, and ZH: project administration. LY: writing – original draft. ZD, HX, and LY: writing – review and editing. All authors have read and agreed to the published version of the manuscript.

## Conflict of Interest

The authors declare that the research was conducted in the absence of any commercial or financial relationships that could be construed as a potential conflict of interest.
